# Corrosion behavior of metastable AISI 321 austenitic stainless steel: Investigating the effect of grain size and prior plastic deformation on its degradation pattern in saline media

**DOI:** 10.1038/s41598-019-48594-3

**Published:** 2019-08-20

**Authors:** A. A. Tiamiyu, Ubong Eduok, J. A. Szpunar, A. G. Odeshi

**Affiliations:** Department of Mechanical Engineering, College of Engineering, University of Saskatchewan, 57 Campus Drive, Saskatoon, S7N 5A9 Saskatchewan Canada

**Keywords:** Mechanical properties, Corrosion

## Abstract

The role of grain size and strain rate on the corrosion behavior of plastically-deformed Ti-stabilized austenitic stainless steel (AISI 321) in saline media was investigated. The as-received coarse-grained alloy (CG: ~37 µm) was subjected to thermomechanical processing to develop fine (FG: ~3 µm) and ultrafine (UFG: ~0.24 µm) grained structures. These samples were deformed under high (dynamic) and low (quasi-static) strain-rate conditions to a similar true strain of ~0.86. Microstructural analyses on specimens after deformation prior to corrosion study suggests a shift from the estimated stacking fault energy value of the steel. Electrochemical tests confirm the highest corrosion resistance for UFG specimens due to the formation of the most stable adsorbed passive film. This is followed by FG and CG specimens in that order. For the three grain sizes, the corrosion resistance of specimen deformed under quasi-static loading condition is higher than that subjected to dynamic impact loading while the corrosion resistance of undeformed samples is the least. This work also confirms the non-detrimental effect of TiCs in AISI 321 austenitic stainless steel on its corrosion resistance. However, TiNs were observed to be detrimental by promoting pitting corrosion due to galvanic coupling of TiNs with their surrounding continuous phase. The mechanism of pitting in AISI 321 in chloride solution is proposed.

## Introduction

Corrosion resistance is an important criterion for selecting materials used in the fabrication of chemical and nuclear plants. This is because the cost of materials degradation due to corrosion and the associated environmental impact are quite substantial^1^. Generally, the composition of austenitic stainless steels (e.g. AISI 304 and other similar grades) is adjusted to meet service requirements in various corrosive environments. Their excellent corrosion resistance is usually hampered by the precipitation of chromium-rich M_23_C_6_ type carbide at the grain boundaries at elevated temperature, thereby promoting intergranular stress corrosion. To inhibit the undesired grain boundary segregation (sensitization), a Ti-stabilized AISI 321 austenitic stainless steel (a derivative of AISI 304) was developed to favor the preferential formation of TiC precipitates, thereby keeping chromium in solution for protection against corrosion^2^. AISI 321 steel is therefore widely used as choice material in the manufacture of heat exchangers for chemical process plants, high-pressure pipes, engine turbines for automobiles and aircraft, and also in nuclear reactors^2–4^.

Several researchers have reported on the corrosion behavior of AISI 321 steel in selected environments that simulate service conditions. For instance, Lv and Luo^5^ studied the effect of low-temperature sensitization (at 380 and 450 °C) on the degree of sensitization (DOS) and corrosion resistance of ultrafine-grained (UFG) AISI 321 steel. The authors observed lower DOS and improved corrosion resistance in the specimen annealed at 450 °C than that annealed at 380 °C. In a separate study^6^, UFG structured AISI 321 steel developed via thermomechanical processing exhibited lower corrosion resistance than coarse grain (CG) specimens in 0.1 M NaCl solution at room temperature. However, the UFG specimen was more resistant to corrosion than the CG specimen in a 0.5 M H_2_SO_4_ solution at room temperature. Moura *et al*.^7^ investigated the influence of stabilization heat treatments on the intergranular corrosion resistance of AISI 321 steel at high temperatures to promote TiC precipitation. Preservation of Cr in solid solution was observed before subsequent use for high temperature (600–800 °C) applications. These researchers reported 950 °C as the optimum stabilizing temperature that inhibits sensitization. Leban and Tisu^8^ reported the deteriorating effect of TiN and deformation-induced martensite (DIM) on the corrosion behavior (pitting and stress corrosion cracking) of cold worked outer exhaust sleeve made of an AISI 321 steel. Other works on the corrosion behavior of AISI 321 and other stainless steels are reported in refs^9–11^ and refs^12,13^, respectively.

The role of grain size, prior deformation, and strain rate on the degradation pattern of AISI 321 stainless steel in corrosive environments are not exhaustively covered in the literature. This is the motivation for the present study. In this work, we have optimized the mechanical properties of the as-received coarse-grained AISI 321 austenitic stainless steel by developing fine and UFG structures in the steel via thermomechanical processing. It, therefore, becomes pertinent to investigate if the unique corrosion resistance of this steel has deteriorated as a result of the grain refinement process. Similarly, the AISI 321 steel with coarse, fine, and ultrafine grain structures were exposed to external loads using quasi-static and dynamic impact loading that can simulate the different loading conditions the steel can experience in service. The aim is also to ascertain how these loading conditions affect the corrosion behavior of AISI 321 steel.

## Material and Methods

AISI 321 metastable austenitic stainless steel (MASS) with nominal composition (in weight %) of 17.61 Cr, 9.17 Ni, 1.56 Mn, 0.42 Mo, 0.40 Si, 0.36 Ti, 0.30 Cu, 0.15 Co and 0.044 C was studied. This steel was received in hot-rolled condition. The alloy is stabilized with titanium and has FCC structure and low stacking fault energy. Cylindrical specimens of 4 mm (diameter) × 4 mm (length) were machined along the rolling direction (RD) for compression tests under both quasi-static and dynamic loading conditions. The quasi-static compression test was conducted using Instron R5500 mechanical testing system with a maximum load cell of 150 kN at a true strain rate of 4.4 × 10^−3^ s^−1^. The dynamic impact test was conducted using the split Hopkinson pressure bar (SHPB) system at a true strain rate of 8.8 × 10^3^ s^−1^. Specimens were compressed under both dynamic and quasi-static loading conditions to a total true strain of approximately 0.86. More details on the SHPB system and the stress wave equations used to generate engineering stress, strain, and strain rate can be found in ref.^14^. The compression direction is parallel to RD, and all compression tests were conducted at ambient temperature.

Metallographic preparation of specimens was conducted using electrolytic polishing with the mixture of 35% sulfuric acid, 45% orthophosphoric acid and 20% de-ionized water as the solution. Scanning electron microscopy (SEM), energy dispersive spectroscopy (EDS) and electron backscatter diffraction (EBSD) measurements were carried out using a SU 6600 Hitachi Field Emission SEM that is coupled with EDS (Oxford X-Max Silicon Drift) and EBSD (Oxford Instruments Nordlys Nano EBSD) detectors. The post-processing of EBSD raw data was conducted using the Oxford Instrument’s Channel 5 post-processing software. The volume fraction of αʹ-martensite in the deformed specimens were measured using Feritscope MP30E. The actual volume fraction of martensite is obtained by multiplying the feritscope reading by a correction factor of 1.7^15^. All tests and analyses were conducted on the plane that is perpendicular to the RD (ND-TD plane) for undeformed specimens and on the plane that is perpendicular to the compression direction (compression plane) for the deformed specimens.

**Figure 1:**
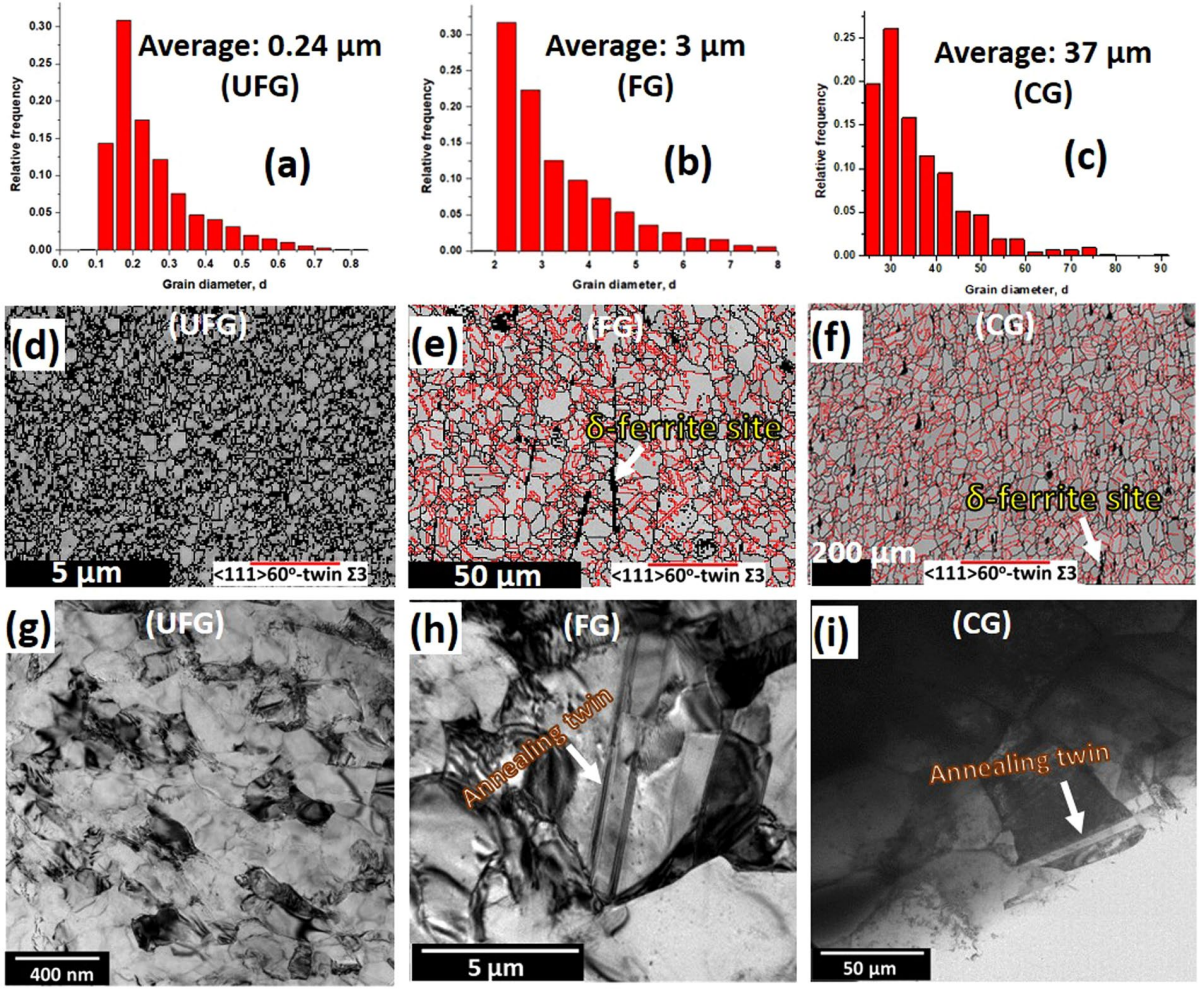
(**a**–**c**) Grain size distribution estimated from large scan area, (**d**–**f**) EBSD band contrast maps with twins and (**g**–**i**) TEM micrographs of the undeformed specimens.

**Figure 2:**
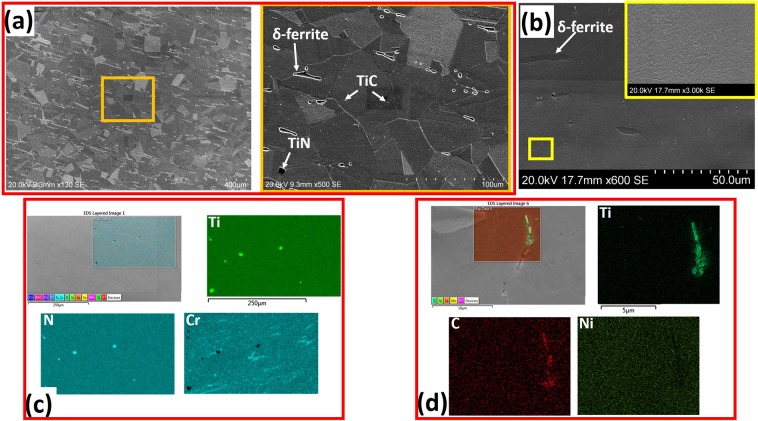
SEM micrographs of (**a**) CG and (**b**) UFG undeformed specimens. EDS maps showing the presence of (**c**) TiN particles and δ-ferrite, and (**d**) TiC particles in the undeformed specimen.

**Figure 3:**
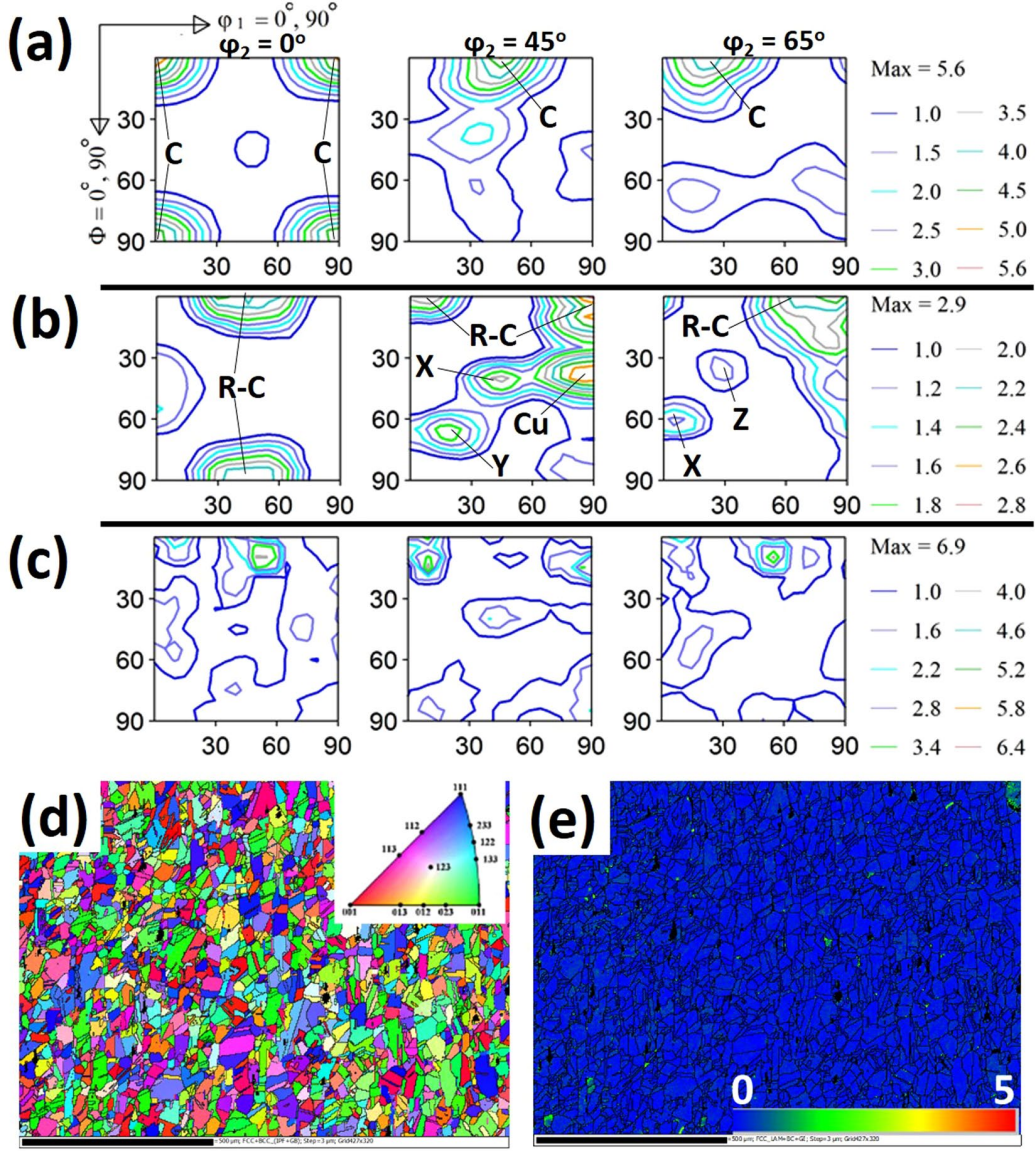
Selected ODF ɸ_2_ sections from the XRD measurement of the undeformed (**a**) UFG, (**b**) FG and (**c**) CG: C {001} <100>, R-C {001} <110>, Cu {112} <111>, X {112} <012>, Y {332} <123>, Z {123} <013>. EBSD (**d**) IPF and (**e**) KAM maps of undeformed CG specimen.

The average grain size of the as-received steel on the ND-TD plane is 37 µm (Fig. 1c). This is hereafter referred to as coarse-grained (CG). A thermo-mechanical process that involved annealing of 50% cryo-rolled as-received CG sample at 750 °C for 10 minutes, and 800 °C for 360 minutes, developed 0.24 (Fig. 1a) and 3 μm (Fig. 1b) grain sizes, respectively. These are hereafter referred to as ultrafine-grained (UFG) and fine-grained (FG), respectively. The details of the thermomechanical processing are provided in ref.^16^. The Vickers hardness values of the undeformed UFG, FG and CG samples are 490, 267 and 186 HV, respectively. The EBSD maps (Fig. 1d–f) and TEM micrographs (Fig. 1g–i) show that the annealing twins are highly suppressed in UFG specimen, but the area fraction of annealing twin in the microstructure increases with an increase in grain size. This observation also suggests that deformation twinning will be difficult in UFG specimen on exposure to external load due to spatial restriction effect. Figure 1e,f also show the presence of intergrain δ-ferrite stringers. δ-ferrite is an intermetallic compound whose formation is promoted by high Cr content in alloys^17^. SEM micrographs also show the presence of the δ-ferrite in CG (Fig. 2a) and UFG specimens (Fig. 2b), and two variants of Ti precipitates, namely, TiN and TiC. While the EDS maps in Fig. 2c confirm the presence of TiN and Cr-rich stringers of δ-ferrite, Figs 2d and S1 in supplementary information affirm the presence of nano-sized (~100 nm) TiC particle in the undeformed specimen. The TiC in austenitic stainless steels satisfy two purposes: first, their precipitation limits the formation of chromium-rich M_23_C_6_ type carbide at the grain boundaries and also prevents intergranular stress corrosion. Secondly, if TiC carbide precipitates as a fine dispersion in matrices and grain boundaries, they improve tensile and creep strength significantly at both high and low temperatures^2^

The selected X-ray diffractometry (XRD) orientation distribution function (ODF) ɸ2 sections show that the undeformed UFG specimen is significantly textured around C {001} <100> orientation (Fig. 3a). The development of R-C {001} <110>, Cu {112} <111>, X {112} <012>, Y {332} <123> and Z {123} <013> orientations are recorded in undeformed FG specimen (Fig. 3b). The intensities of these orientations decrease as the grain size increases from fine to coarse (Fig. 3c). Figure 3d shows the EBSD IPF color map that reveals random orientation of the undeformed CG specimen and its corresponding very low Kernel average misorientation (KAM) value map (Fig. 3e), typical of the KAM of UFG and FG specimens. KAM measures local misorientation and it can indicate strain distribution in a deformed microstructure. Low KAM value depicts low accumulated strain and vice-versa.

All electrochemical corrosion tests on electropolished specimens were conducted using a potentiostat (Interface 1000, Gamry Instruments) as schematically shown in Fig. S2. Measurements were carried out in the order: open circuit potential (*E*_oc_), electrochemical impedance spectroscopy (EIS) and Tafel polarization, using a three-electrode system. A graphite rod was utilized as the counter electrode while an Ag/AgCl (sat. KCl) reference electrode measured the magnitudes of electrical potentials within this work. These electrodes were connected to the potentiostat with the deformed or undeformed specimens as the working electrodes in parallel. Before the test, a 30-minute equilibration time was allowed for each metal coupons exposed to the aerated corrosive solution (3.5 wt.% NaCl) at a defined geometric area (0.35 cm^2^). EIS measurements were conducted by applying a 10-mV sinusoidal alternate low-voltage perturbation (peak-to-peak) with the frequency ranging from 10^4^ to 10 mHz, 10 points per decade at *E*_oc_. The Tafel polarization test was also conducted after the impedance measurements by applying an overpotential of ±250 mV from *E*_oc_ at a scan rate of 0.5 mV s^−1^. The surface morphologies of each metal surface after exposure to NaCl corrosive solutions were examined using SEM. SEM analyses complemented the electrochemical corrosion tests for both deformed (under both dynamic and quasi-static conditions) and undeformed specimen. Inherent pitting episodes represent a measure of resistance to chloride-induced corrosion by each specimen within the duration of the test. All the electrochemical measurements presented in this study are reproducible; results are representative of the multiple measurements.

## Results and Discussion

### Mechanical (dynamic and quasi-static) behavior of AISI 321 austenitic steel

**Figure 4:**
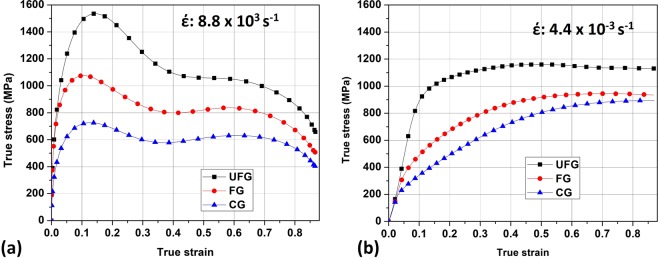
True stress-strain curves of specimens deformed under (**a**) dynamic and (**b**) quasi-static loading conditions.

True stress-strain curves depicting the dynamic impact response (8.8 × 10^3^ s^−1^) and the quasi-static compressive behavior (4.4 × 10^−3^ s^−1^) of AISI 321 steel are presented in Fig. 4a,b, respectively. At both strain rates, the magnitude of yield strength increases as the grain size decreases. However, a significant difference between the dynamic and quasi-static stress-strain curves exist in the deformation process in the plastic region. At the start of the plastic region, the dynamic curve (Fig. 4a) falls; it rises to the maximum flow stress and fall again. This trend could be attributed to the rigorous competition between the strain-hardening (rising) and thermal softening (falling) of the impacted specimens. The strain hardening could be attributed to activation of different deformation and/or strengthening mechanisms. Meanwhile, thermal softening leads to thermo-mechanical instability and loss of load-carrying capability as the temperature of the specimen is raised during the dynamic deformation process. The temperature rise in the specimen is due to the conversion of the 90% of the kinetic energy of projectile to thermal energy during high strain rate^18^. On the other hand, the quasi-static curves in Fig. 4b shows a consistent rise (strain-hardening) up to the final deformation true strain of ~0.86.

### Microstructural evaluation before corrosion test

#### Operational deformation mechanisms and macrotexture evaluation

**Figure 5:**
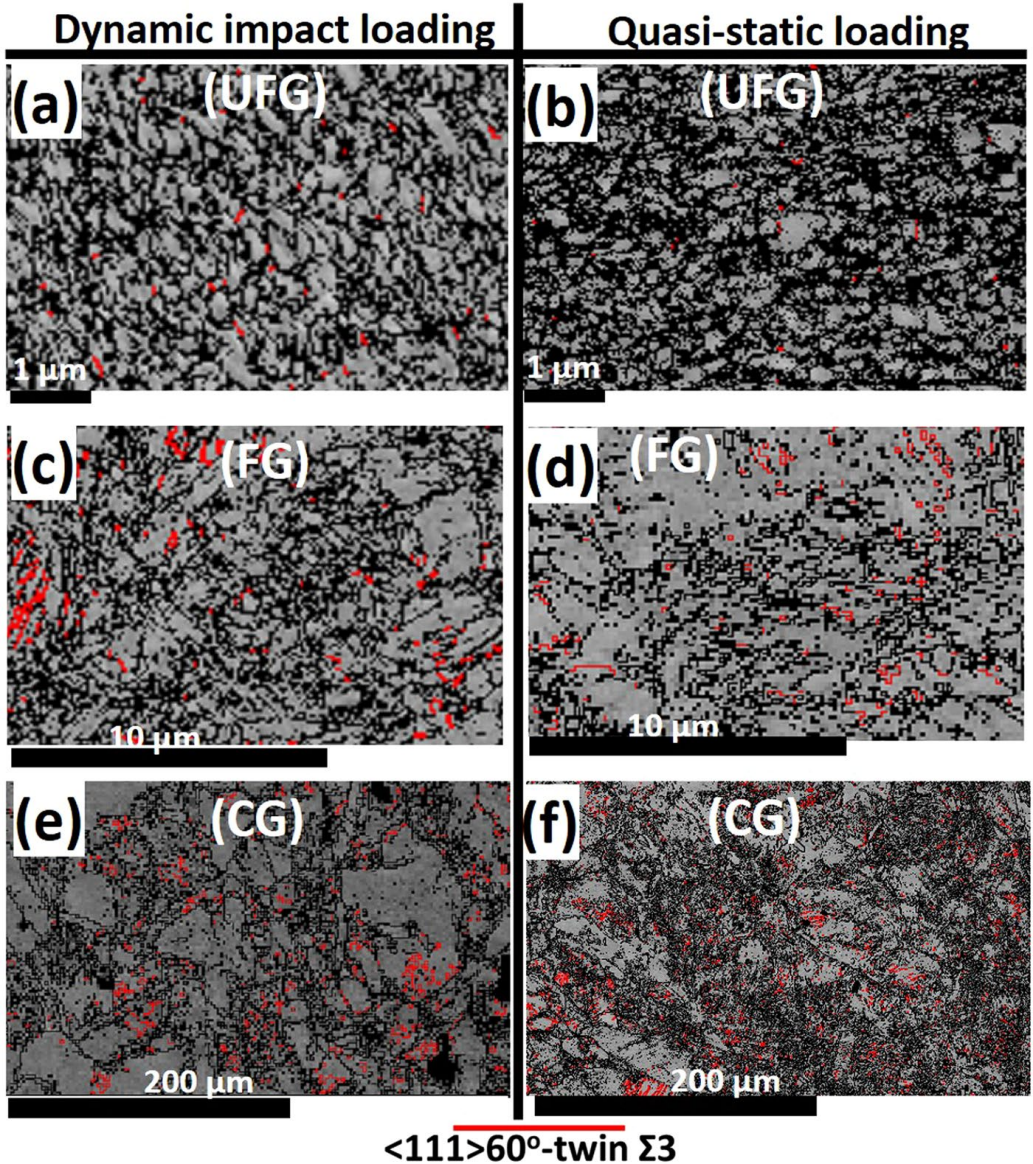
EBSD band contrast and twin maps for specimens subjected to (**a**,**c**,**e**) dynamic and (**b**,**d**,**f**) quasi-static loading conditions.

The EBSD band contrast and twin maps in Fig. 5 confirms the occurrence of deformation twinning and also the role of grain size and strain rate on twinning activity. These maps reveal near-absence of twins in UFG specimens and its fraction increases with increase in grain size. Similarly, relatively lower twin area fraction is recorded in specimens deformed under dynamic loading conditions (Fig. 5a,c,e) than those compressed under quasi-static condition (Fig. 5b,d,f). This implies that both coarse grain size and low strain rate favors more twinning activities. Under tensile load, Ueji *et al*.^19^ observed that the evolution of deformation twinning becomes more difficult as the grain size decreases in a high Mn austenitic TWIP steel. A similar result was also observed in the finite element simulation of AISI 304 ASS under uniaxial tension^20^.

**Figure 6:**
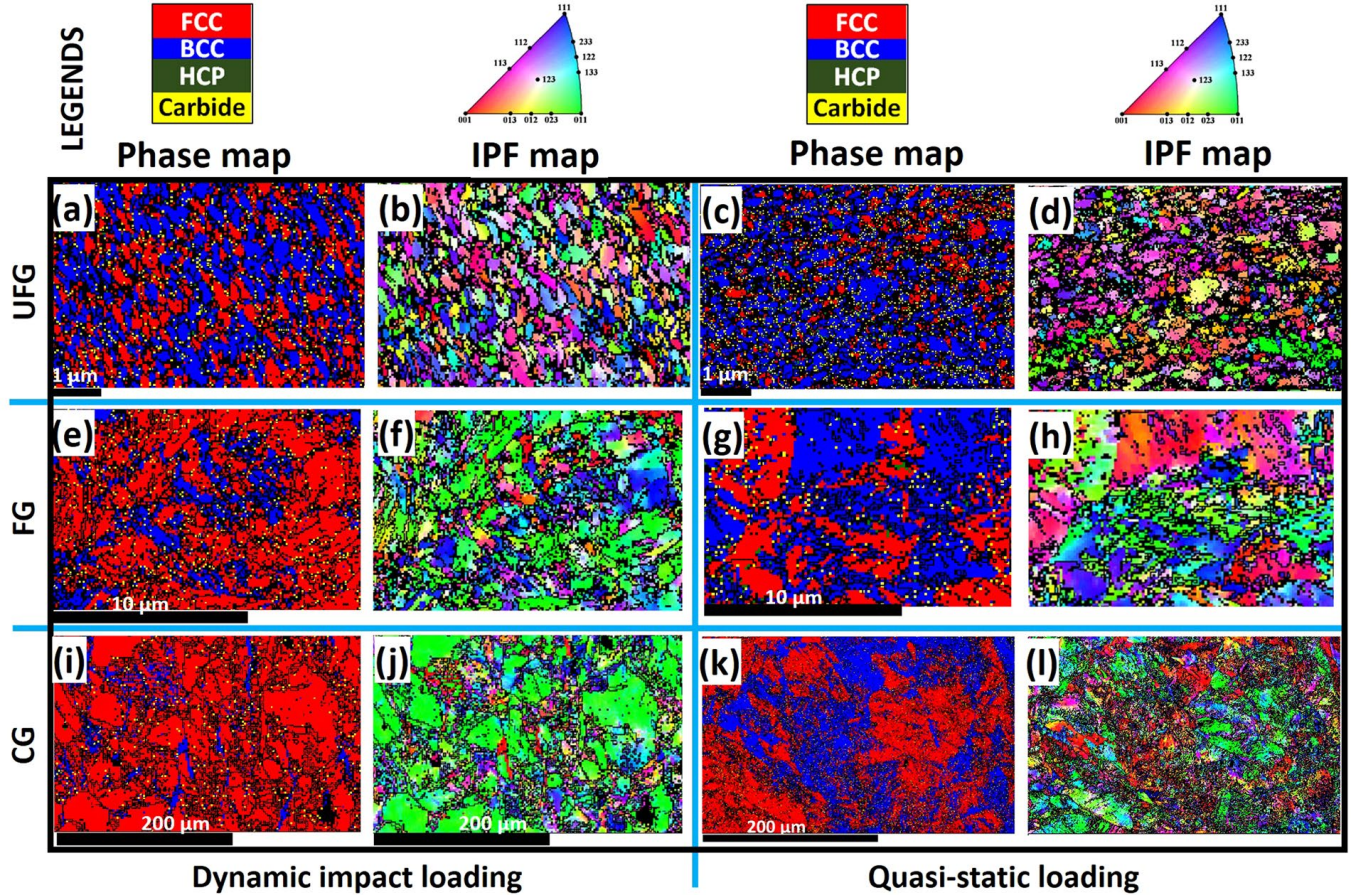
EBSD (**a**,**c**,**e**,**g**,**i**,**k**) phase and (**b**,**d**,**f**,**h**,**j**,**l**) IPF maps for specimens deformed under dynamic and quasi-static loading.

The EBSD phase maps in Fig. 6(a,c,e,g,i,k) also indicate the occurrence of deformation-induced martensitic transformation and its variability with grain size and strain-rate. At both high (a, e, i) and low (c, g, k) strain-rates, the area fraction of α′-martensite (blue) decreases with an increase in grain size. The higher fraction of α′ in UFG specimen could be largely due to the presence of higher triple junctions (a potential α′ nucleation sites) than those of fine and coarse specimens. The formation of DIM at triple junction of grain boundaries are also reported in deformed AISI 304 LN austenitic stainless steel, a derivative of AISI 321^21^. The dependence of martensitic phase transformation on grain size is also reported in another previous work^21^. Similarly, a higher fraction of α′ martensite was recorded in specimens deformed under quasi-static compressive load than their dynamic-impacted counterpart. The lower fraction of α′ in the specimen under dynamic impact loading is due to temperature rise in the specimen that suppresses phase transformation. The corresponding IPF maps of the phase maps are presented in Fig. 6(b,d,f,h,j,l). While the stable end-orientation of the deformed austenite phase is CD||[110] with a minor spread near CD||[111] fibre texture, that of the DIM is near CD||[100]. Similar texture results have been reported in a cold-rolled AISI 304L stainless steel^22^.

**Figure 7:**
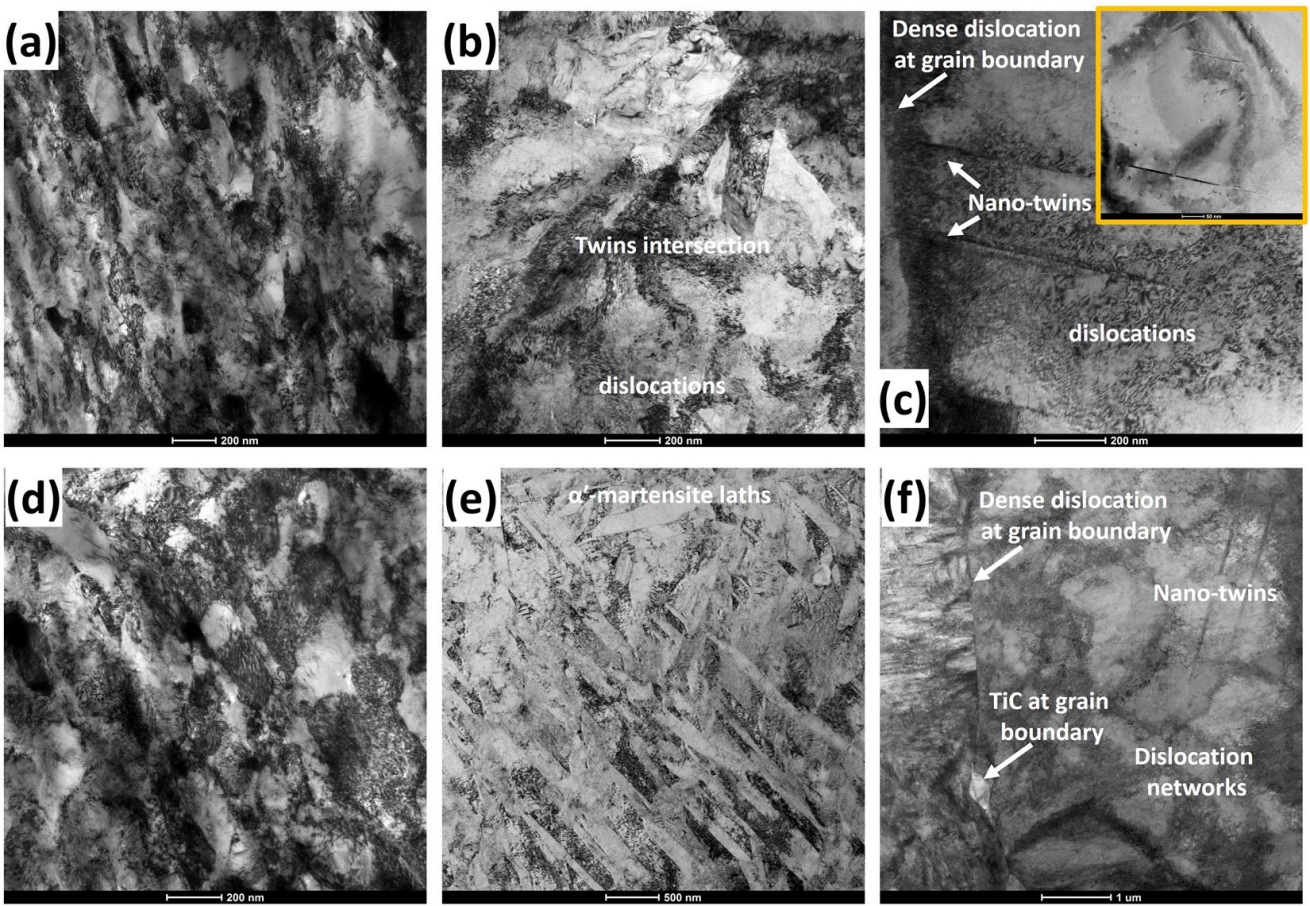
TEM bright field micrographs of specimens subjected to (**a**–**c**) dynamic and (**d**–**f**) quasi-static loading: (**a**,**d**) UFG and (**b**,**c**,**e**,**f**) CG specimens.

**Figure 8:**
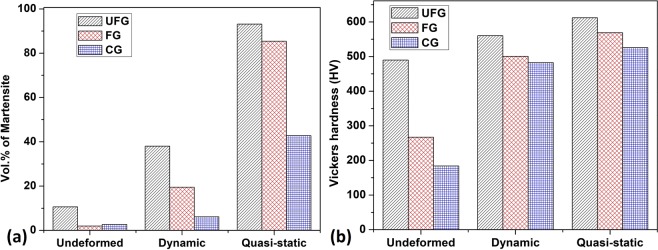
(**a**) Volume % of deformation-induced martensite and (**b**) Vickers hardness of undeformed and deformed specimens.

The activated deformation mechanisms earlier observed using the EBSD technique are further verified by the TEM results in Fig. 7. For brevity, only the TEM micrographs of the UFG (Fig. 7a,d) and CG (Fig. 7b,c,e,f) specimens are presented. Under both dynamic and quasi-static loading conditions, TEM micrographs affirm near-absence of deformation twin in the UFG specimens. The TEM micrographs of the CG specimens under dynamic loading condition reveal the formation of deformation twin from the grain boundaries (Fig. 7b,c) and within the grain (inset in Fig. 7c) at regions of high dislocation densities or networks. Meanwhile, CG specimens compressed under quasi-static condition exhibit massive deformation-induced martensitic transformation (Fig. 7e) in addition to deformation twinning and slip (Fig. 7f). The results of the feritscope measurement of α′ fraction in the deformed specimens affirm that the decrease in both grain size and strain rate leads to an increase in α′ fraction (Fig. 8a) and to a corresponding increase in hardness (Fig. 8b). From these observations, it can therefore, be safely concluded that hardening in metastable AISI 321 stainless steel originates from multiple sources. In addition to grain boundary strengthening, hardening in AISI 321 steel is attributed to the occurrence of deformation twinning acting as a barrier to dislocation motion, deformation-induced martensitic transformation, dislocation multiplication during slip, and precipitation of carbides that act as barriers to dislocation motion during plastic deformation.

#### Plausible effect of grain size and strain rate on stacking fault energy

**Figure 9:**
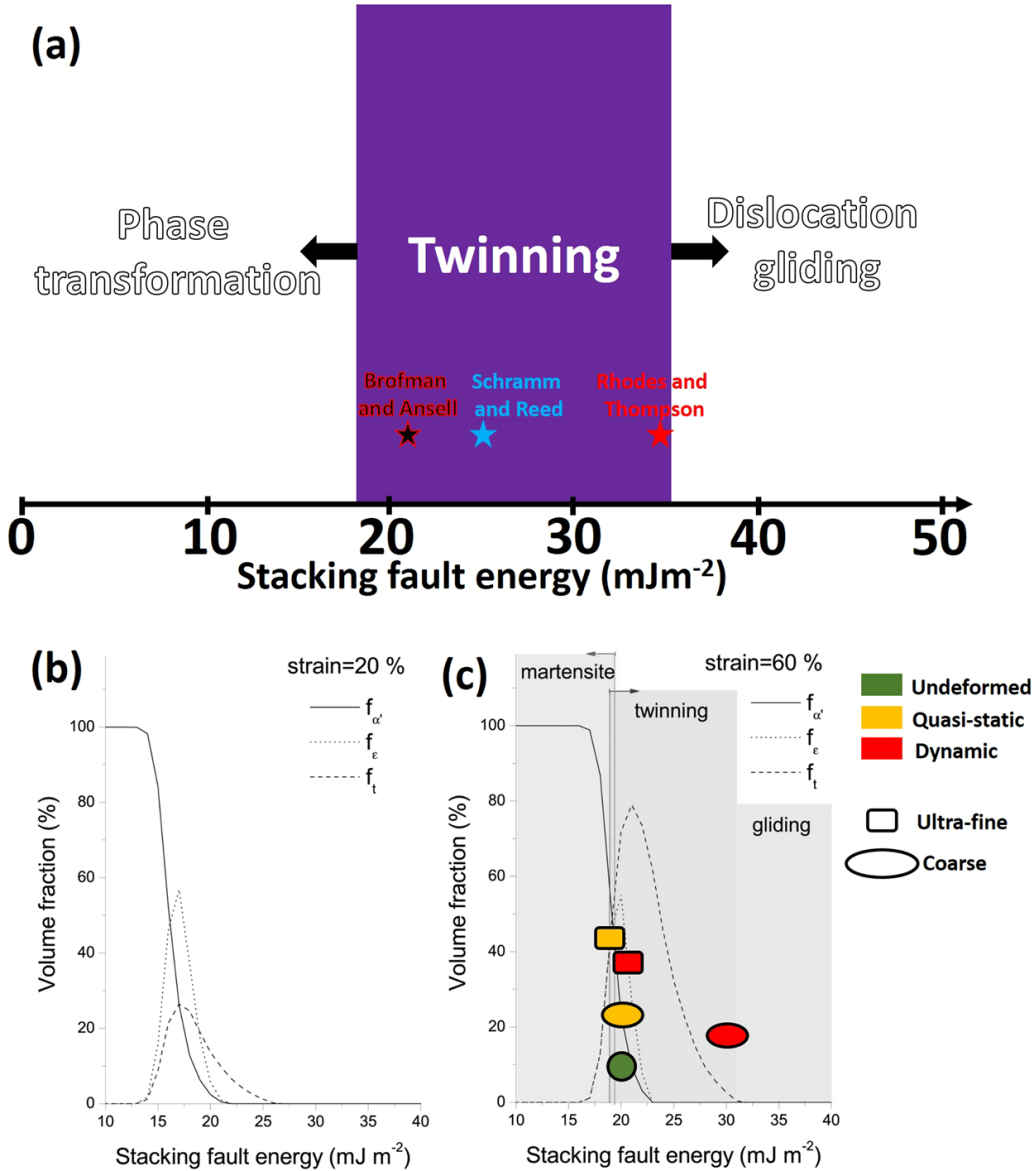
(**a**) Schematic showing partitioned SFE and their corresponding activated deformation mechanism, (**b**,**c**) effect of strain on the volume fraction of DIM and twinning for different stacking fault energies^30^.

The plausible variation of stacking fault energy (SFE) with grain size and strain rate can be postulated from the mechanical behavior and microstructural evolution in the deformed UFG, FG and CG specimens. Generally, the operational deformation mechanisms in a metal are strongly influenced by SFE^23^ and are partitioned in such a manner that martensitic phase transformation, twinning, and slip dominates when the SFE are <18 mJm^−2^, in the 18–35 mJm^−2^ range and above 35 mJm^−2^, respectively^24^, as schematically shown in Fig. 9a. However, regardless of the activated deformation mechanisms, plastic deformation occurs by slip^25^. Although *γ*-SFE depend on factors such as chemical composition and temperature^26^, the existing compositional equations (Eq. 1: Brofman and Ansell^27^, Eq. 2: Schramm and Reed^28^ and Eq. 3: Rhodes and Thompson^29^) for estimating SFE does not take into consideration the role of grain size. However, our experimental result suggests a possible variation with grain size.

1 $$\gamma =16.7+2.1( \% Ni)-0.9( \% Cr)+26( \% C)$$

2 $$\gamma =-53+6.2( \% Ni)+0.7( \% Cr)+3.2( \% Mn)+9.3( \% Mo)$$

3 $$\gamma =1.2+1.4( \% Ni)+0.6( \% Cr)+17.7( \% Mn)-44.7( \% Si)$$

The estimation of SFE for AISI 321 from these equations (Eqn. 1: 21 mJm^−2^, Eqn. 2: 25 mJm^−2^ and Eqn. 3: 34 mJm^−2^) could, therefore be assumed for the undeformed CG sample as indicated in Fig. 9a. Since both deformation induced martensitic transformation and twinning can readily occur in AISI 321 steel, it is suggested that Brofman and Ansell equation gives a better approximation (~21 mJm^−2^) of SFE for CG AISI 321 steel, though, the equations do not consider all elements. This is simply because at ~21 mJm^−2^, DIM and twinning can still operate jointly, as it can be observed in Figs 5–7. This was also reported in AISI 304 austenitic stainless steel which is an intermediate SFE metal^30^. It should be noted that AISI 304 is a derivative of the investigated AISI 321 in the current study. Elsewhere^31^, Brofman and Ansell equation has been reported and confirmed to show the best correlation with experimental results in metastable austenitic stainless steel.

The variation of dominant deformation mechanisms that is largely due to the effect of grain size and strain rate (Figs 5–7) however suggests that the SFE of AISI 321 steel possibly deviates from the estimated value of 21 mJm^−2^. In a metastable stainless steel, Galindo-Nava and Rivera-Díaz-del-Castillo^30^ established the variation of SFE with strain at a fixed strain rate of 10^−3^ s^−1^ as presented in Fig. 9b,c. However, it is thought that SFE possibly shifts slightly to the left (i.e. decreases) due to compressive loading at low strain rate and a shift to the right (i.e. increases) at high strain rate. This is premised on the observed substantial promotion and suppression of DIM formation at low and high strain rates, respectively. The suppression of DIM at high strain rates is due to adiabatic heating during deformation. Adiabatic heating has been reported to result in increased SFE^32^. Similarly, the refinement of CG specimen to UFG structure possibly led to a decrease in SFE as DIM was highly promoted and twinning was highly suppressed in compressed UFG specimens as indicated in Fig. 9c. For instance, UFG specimen (Fig. 6) underwent extensive deformation-induced martensitic phase transformation that is characteristic of alloys with SFE below 18 mJm^−2^ than those of CG specimens. On the other hand, the deformation-induced twinning occurred more readily in CG specimens (Fig. 5), which is characteristic of alloys with SFE in the 18–35 mJm^−2^ range, than those of UFG specimens.

### Corrosion test results

The corrosion behaviors of both undeformed and deformed (dynamic and quasi-static) specimens with different grain sizes were investigated using corrosion electrochemistry and surface analysis after exposure to 3.5 wt.% NaCl solution. Changes in corrosion resistance of this Ti-stabilized AISI 321 austenitic stainless steel after the enhancement of its mechanical properties via grain refinement are discussed in this section.

#### Effect of grain size on corrosion resistance

**Figure 10:**
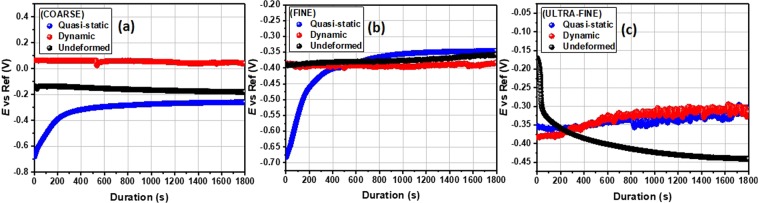
*E*_oc_ variation with time for undeformed and deformed (under dynamic and quasi-static loading conditions) specimens with varying grain sizes in 3.5 wt.% NaCl solution at room temperature.

Since electrochemical tests are conducted at open circuit potential (*E*_oc_), the *E*_oc_ vs. time curves for the specimen in NaCl were generated (Fig. 10). There is no defined trend in the *E*_oc_ data among specimens of different grain sizes. The observed results in Fig. 10 are also independent of the strain rate (dynamic or quasi-static) within the duration of the test. The *E*_oc_ vs. time curves for the CG and FG specimens deformed under quasi-static condition show a steady rise in *E*_oc_ between 0 and 400 seconds, then normalized afterward at −0.30 and −0.35 V, respectively. Except for the FG specimens, the CG and UFG specimens reveal more positive *E*_oc_ values under dynamic loading condition, especially between the mid and end of the test. For CG and FG specimens, the *E*_oc_ vs. time curves for undeformed and deformed (high strain rates only), are parallel to each other between 0.10 and −0.20 V and between −0.35 and −0.40, respectively. The trend in the magnitude of *E*_oc_ suggest different electrochemical behaviors for specimens deformed at both strain rates under compression, independent of their grain sizes. These curves indicate significant surface responses within the duration of the test, denoting that 30 mins is sufficient to attain a steady-state condition with few fluctuations in the media.

**Figure 11:**
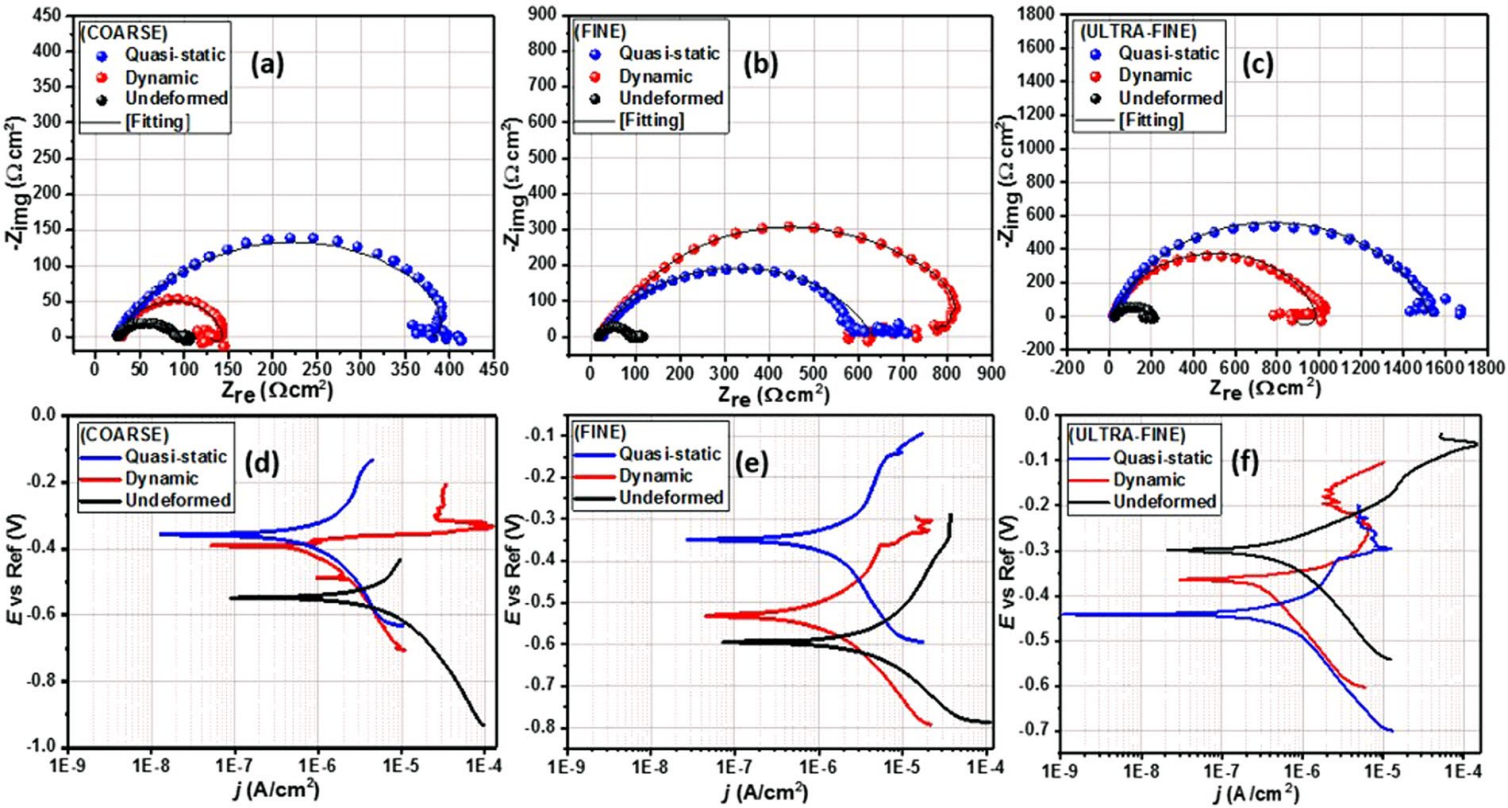
Nyquist (**a**–**c**) and Tafel polarization (**d**–**f**) curves for stainless-steel substrates with coarse, fine and ultra-fine grain sizes under deformation at different strain rates as well as their undeformed counterpart exposed to 3.5 wt.% NaCl solution at room temperature.

**Figure 12:**
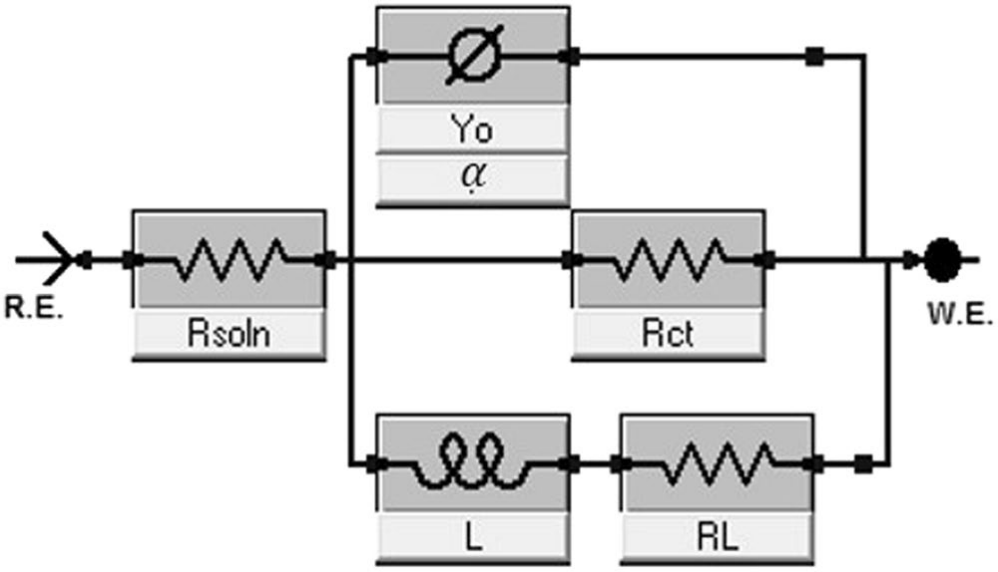
Equivalent circuit model utilized in fitting the experimental impedance data for both undeformed and deformed stainless-steel substrates with varying grain sizes.

The electrochemical impedance spectroscopy (EIS) was used to determine the corrosion resistance of these specimens directly by measuring the electrical parameters related to the resistance against chloride ion ingressions. The results from this test provide data about the electrode processes based on the electrochemical responses. Figure 11(a–c) presents the Nyquist spectra for both undeformed and deformed specimens with varying grain sizes exposed to 3.5 wt.% NaCl solution. These impedance curves reveal similar corrosion pattern for all specimens since their electrochemical features are relatively similar. At high frequencies, these curves are characterized with single capacitive loops due to inherent charge transfer processes controlling the corrosion reactions^33^. The presence of some unresolved inductive-type loops is also conspicuous at lower frequencies due to relaxation of diffused or adsorbed species. The observed unevenness in the impedance curves could be linked to the consequence of corrosive attack upon exposure of the metal surfaces to corrosive ions and molecules within the saline media. This could also be associated with other phenomena leading to the prevalence of micro-roughness and surface heterogeneities on the stainless-steel electrodes^33,34^. Since the corrosion resistance of these specimens could be a function of the size of impedance capacitive loops^35^, we then could deduce that specimens corresponding to wider Nyquist curve diameters are more resistant to chloride-induced corrosion. By physical inspection, it could be inferred that both deformed and undeformed UFG specimens are more resistant to corrosion than those of the FG and CG specimens. By far, CG specimens trail behind as one with the least resistance to corrosion, whether deformed or not. Experimental impedance data were fitted into an appropriate equivalent circuit model as shown in Fig. 12. The electrochemical parameters extracted from this theoretical operation are presented in Table S1. Good fittings were achieved with relatively small chi (*χ*^2^) square values to support this claim. The circuit model consists of elements representing solution resistance (*R*_soln_), charge transfer resistance (*R*_ct_), inductive resistance (*R*_L_), double layer capacitance (*Q*_dl_) and an inductor (*L*). The observed magnitude of *R*_ct_ in Table S1 follows the order: UFG > FG > CG, for both undeformed and deformed specimens. *R*_ct_ values for CG specimens are determined to be the least, and are 83, 125, 401 Ω for the undeformed, dynamic and quasi-static deformed specimens, respectively. The values of this parameter increased markedly for those with ultra-fine grains and are 175 Ω (undeformed), 1081 Ω (dynamic), 1509 Ω (quasi-static). Since charge transfer resistance (*R*_ct_) represents the opposition to the flow of ionic currents, the trend in this parameter for AISI 321 stainless steel with the three grain sizes under study suggests the formation of a more stable adsorbed passive film for UFG specimens.

In all, more enhanced corrosion resistance was observed for UFG specimens under quasi-static loading condition while the CG specimen corroded more compared to the rest of the specimens. The magnitude of capacitive (*Q*_dl_) can account for water uptake within the duration of the corrosion test. The trend of its values is the reverse of that of *R*_ct_ for the three grain sizes investigated; higher values were obtained for undeformed CG specimens. *Q*_dl_ values for undeformed CG, FG and UFG specimens are 68.1, 36.3 and 48.4 µF cm^−2^ s^−(1−αc)^, respectively. Lower values of *Q*_dl_ denotes reduced water uptake by the adsorbed passive film and markedly describes the barrier performance of the film. The capacitive element within the circuit design is constant phase elements (CPE, *Q*) deployed to ensure precise fitting results while also accounting for inherent metal surface inhomogeneity. The impedance of CPE could be defined as expressed in Eq. 4. In this equation, *Y*_*o*_ and *α* represent the frequency independent factors while *ω* is the angular frequency (2 *π*f, measured in rad/sec) of the ac voltage applied to the electrolytic cell. The magnitude of *α* is affected by surface inhomogeneities and gross roughness. For an ideal surface, this quantity reaches unity (1) when *Z*_*CPE*_ is considered a pure capacitor. In this work, the values of *α* are close to unity.

4 $${Z}_{CPE}={[{Y}_{o}{(j\omega )}^{\alpha }]}^{-1}$$

Corrosion studies with EIS technique were complemented by Tafel polarization test in the same saline solution. Polarization curves for the steel specimens with varying grain sizes under different strain rates as well as those of their undeformed counterparts are presented in Fig. 11(d–f). After curve fitting, the polarization parameters were derived by extrapolation of the linear portions of the anodic and cathodic sections of the curves, and listed in Table S1. These parameters consist of corrosion current density (*j*_corr_), corrosion potential (*E*_corr_), anodic (*β*_a_) and cathodic (*β*_c_) Tafel slopes. The dissolution of the alloy specimens is characterized by changes in the values of *E*_corr_ and *j*_corr_. Evidence of passivation is observed for corrosion resistant specimens due to the formation of adsorbed stable passive films consistent with Ti-stabilized austenitic stainless steel. This is conspicuous for UFG specimens irrespective of the deformation mode. The prior deformation at low and high strain rate resulted in passivation at an *E*_passivation_ of −0.30 V (both strain rate) for UFG specimens while that of the FG specimens were recorded at −0.15 V (low strain rate) and −0.35 V (high strain rate), respectively, and −0.3 V (both strain rate) for the CG specimens. The observed surface passivation is also consistent with decreasing magnitude of *j*_corr_ for FG specimens compared to those with coarse grains. The magnitudes of *j*_corr_ for ultra-fine specimens under quasi-static, dynamic, and undeformed conditions in NaCl solution are the lowest, and are 0.29, 0.59, and 16.30 µA/cm^2^, respectively. Higher values of *j*_corr_ were obtained for CG specimens independent of the rate of deformation. These were determined to be 0.43, 16.6, and 142.9 µA/cm^2^ for specimens deformed under quasi-static and dynamic loading conditions and for undeformed specimen, respectively.

In the undeformed and dynamic loading conditions, the magnitudes of *E*_corr_ for the FG and CG specimens are more negative compared to those of UFG specimens. For instance, the *E*_corr_ values for both FG and CG specimens deformed under dynamic loading condition were −0.53 and −0.39 V, respectively, while −0.36 V was recorded for UFG specimen. On the other hand, −0.35 V was recorded for both FG and CG specimens deformed under quasi-static condition. The improvement in the corrosion resistance of stainless steels due to stronger stability, more compactness, lower defect density and higher chromium content of passive films developed on the nano-crystalline structure in different corrosive media compared to the conventional coarse grain structure has been reported elsewhere^36^. Miyamoto^37^ also reported that UFG structure exhibits lower passive current and higher breakdown potential in chloride-containing media, which translates to higher corrosion resistance. Ralston *et al*.^38^ revealed the existence of a relationship between corrosion rate and grain size (Eqn. 5) that is analogous to the classical Hall-Petch relation as follows:

5 $${j}_{corr}=(A)+(B)g{s}^{-0.5}$$

where A is a constant and a function of the environment (corrosive media). B represents a material constant, which depend on the composition or impurity level of the material. Ralston *et al*. concluded that UFG structures would be more corrosion resistance if the grain boundary density dictates oxide-film conduction rate on substrate’s surface of low to passive corrosion rates (i.e. *j*_*corr*_ < 10 µAcm^−2^). However, when the dissolution rates are higher than 10 µAcm^−2^ (i.e. in the absence of oxide film), increase in grain boundary densities (grain refinement) will enhance the overall surface reactivity and in turn, increase corrosion rate.

Although corrosion resistance of stainless steels is improved by grain refinement to nano-structures, processing routes and parameters used to fabricate these nanocrystalline structures have a strong effect on their corrosion behavior^36^. UFG/Nanocrystalline structures produced by techniques such as sputtering, thermomechanical processing (cold-rolling and annealing), equal channel angular pressing (ECAP) and surface mechanical attrition treatment (SMAT) have been reported to be more corrosion resistance than their coarse-grained counterpart^36,38^. This work, therefore, confirms that the development of UFG via thermomechanical processing (cryo-rolling and annealing) improves corrosion resistance.

#### Effect of prior deformation rate on corrosion resistance

**Figure 13:**
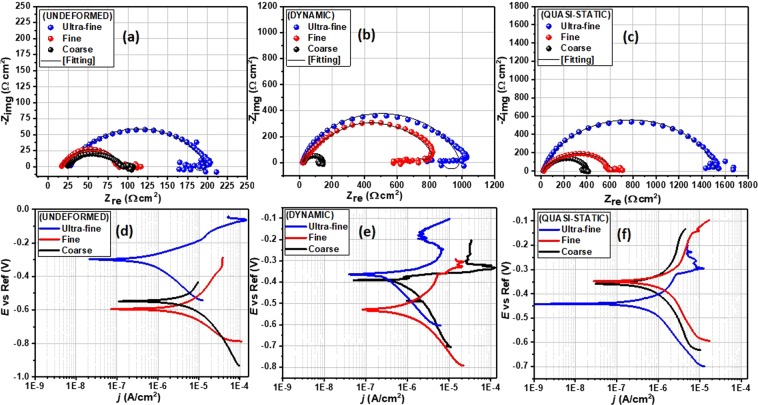
Nyquist (**a**–**c**) and Tafel polarization (**d**–**f**) curves for both undeformed and deformed stainless-steel substrates with varying grain sizes exposed to 3.5 wt.% NaCl solution at room temperature.

The corrosion resistance of AISI 321 MASS has revealed an interesting trend with varying grain sizes. However, since grain refinement has also enhanced its mechanical properties, the extent to which prior deformation and the rate at which the deformation occurred has affected corrosion resistance will also be examined. To accomplish this, the Nyquist curves in Fig. 11 were rearranged to highlight only the effect of strain rates under compression for individual specimen. Figure 13 depicts the Nyquist curves for specimen deformed under dynamic and quasi-static loading conditions compared to their undeformed counterpart. The sizes of the Nyquist semi-circle diameters are wider for more resistive systems. The specimens deformed under quasi-static loading condition are more resistant to corrosion compared to those subjected to dynamic loading condition. By inspection, it could also be inferred that undeformed specimens are less resistant to corrosion; significant amount of corrosion occurs independent of the grain size. *R*_ct_ values for UFG, FG and CG specimens under low strain rate are 1509, 609, 401 Ω, respectively, while those deformed at high strain rate are 1081 Ω (UFG), 822 Ω (FG), 125 Ω (CG). The values of this parameter decrease markedly for undeformed specimens: 175, 91, 83 Ω, in similar order. This implies that the magnitude of *R*_ct_ values for this Ti-stabilized AISI 321 austenitic stainless-steel follows the order: quasi-static > dynamic > undeformed, for all grain sizes, except for FG specimens. The trend in *R*_ct_ and *Q*_dl_ for both undeformed and deformed specimens with varying grain sizes are presented in Table S2.

Metallic systems with significant corrosion resistance continue to show distinct passivation and lower corrosion currents in the polarization curves (Fig. 13d–f). Deformed specimens at low strain rates are characterized by low rate of dissolution in the saline NaCl solution. Lower values of *j*_corr_ are consistent with the formation of stable passive films on the corrosion-resistant steel specimen, especially the deformed UFG specimens. The *j*_corr_ values for the undeformed specimens are consistently higher than those of the deformed specimens. This is a strong indication that prior deformation contributed to the corrosion resistance. For any given grain size, the jcorr values of the specimens deformed under quasi-static loading condition are the least. For instance, the jcorr values for UFG specimens are 16.3 µA/cm^2^ (undeformed), 0.59 µA/cm^2^ (dynamic), and 0.29 µA/cm^2^ (quasi-static). This implies that corrosion resistance follows the trend quasi-static > dynamic > undeformed for all grain sizes. The magnitudes of *E*_corr_ for undeformed and deformed (under dynamic loading conditions alone) UFG specimens are more positive and significantly higher; −0.29 and −0.36 V, respectively. The trend of other electrochemical parameters is displayed in Table S2. In this work, it could be further concluded that deformed specimens (under compression) are resistant to chloride-induced corrosion at ambient temperature compared to the undeformed specimens; a similar observation is reported in ref.^39^. These results suggest that corrosion resistance by surface passivation was enhanced by bulk deformation^40^ mostly at low strain rates which will be further explained by surface analysis using SEM. During the sanding and brushing treatments of AISI 316L, other authors^41^ have also reported an improved corrosion resistance via plastic deformation and compressive residual stress. Phadnis *et al*.^42^ also reported that plastic deformation played a significant role on the thickness of protective oxide film in a cold-rolled AISI 304 austenitic stainless steel in de-aerated 3.5% NaCl solution. These authors found cold-rolling to be beneficial to forming thicker oxide film with higher Cr/Fe ratio on cold-rolled specimen than the unrolled specimen.

Despite the evolution of deformation-induced martensite (DIM), the plausible reason for higher corrosion resistance in deformed specimens (compared to the undeformed) could be texture-related. While the austenite phase in the as-received undeformed specimen has random orientations, the phases in the deformed specimens are significantly textured. As discussed in section 3.2 (Fig. 6), both the deformed austenite phase and the DIM possesses stable-end orientations (e.g. CD||[110] in the former and CD||[100] in the later) that could be more resistant to corrosion in saline media as also observed in another study^43^. Because CD||[110] is the stable end-orientations for compressed FCC metal^44^, it is believed that the stable end-orientation in uniaxial-compressed BCC metals (CD||[111] and [100]^44^) could also be more corrosion resistant than other orientations. Hence, the close-packed crystallographic planes (CD||[110] for austenite and CD||[100] for martensite) nullifies the adverse effect of DIM on the passivation and repassivation features of AISI 321 steel. This is in agreement with observations in a previous study on AISI 304 L stainless steel^22^.

A number of reasons could also be responsible for higher corrosion resistance in specimens subjected to quasi-static compressive load than those deformed under dynamic loading condition. It is clear, as earlier stated, that there is a significant temperature rise in specimens subjected to dynamic impact loading that could influence the intrinsic property of the metal in a way that is different from those subjected to quasi-static loading, which has a negligible temperature rise. From the critical stress for mechanical twinning’s (*σ*_*twin*_) point of view, i.e. ${\sigma }_{twin}=6.14\,(\Gamma /b)$, where *Γ* is the SFE and b is the Burgers vector of the Shockley partials^45^, higher material’s SFE results in higher *σ*_*twin*_ and a lower tendency for twinning, and vice-versa. As described in Fig. 9, the increase in temperature of specimen subjected to dynamic loading could result in an increase in SFE. Therefore, from the *σ*_*twin*_ expression (also confirmed in Fig. 5), the deformation twinning will be more favored in specimens deformed under quasi-static compression and less favored in specimens subjected to dynamic-impact load due to an increase in SFE as a result of temperature rise^46^. Evolution of more deformation twin could therefore, be one of the possible reasons for the better corrosion resistance of specimens deformed under quasi-static condition in comparison with specimen subjected to dynamic impact loading. This is in agreement with the findings of Chen *et al*.^40^, who reported that low-energy twins within the austenite grain of AISI 304 stainless steel suppresses chromium depletion at the grain boundaries and promotes passive film formation. Wang *et al*.^47^ also reported a remarkable decrease in the corrosion rate of Mg-3Al-1Zn due to the activation of high density twins. While deformation twinning is beneficial to improving corrosion resistance, it is necessary to highlight that near absence of twins were observed in both undeformed and deformed UFG specimens. In this case, other factors such as the combined presence of close-packed crystallographic planes in both deformed austenite and DIM (highest in deformed UFG specimens), and the dominance of higher grain boundary density over oxide film conduction rate could be beneficial to improving corrosion resistance in undeformed and deformed UFG specimens.

### Surface morphology

**Figure 14:**
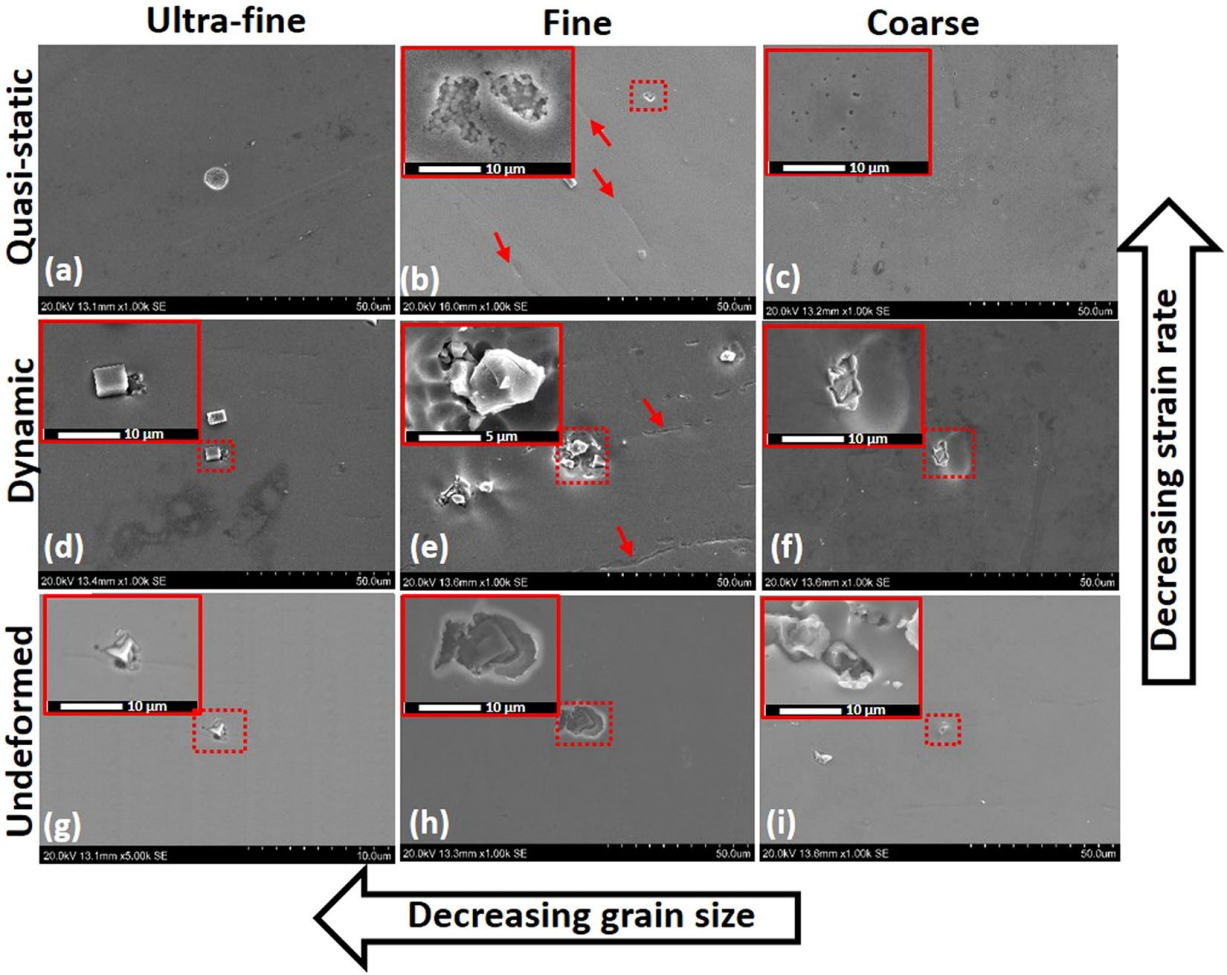
SEM micrographs of stainless-steel substrates with different grain sizes under deformation at different strain rates as well as their undeformed counterpart exposed to 3.5 wt.% NaCl solution at room temperature.

The results of electrochemical corrosion investigations of CG, FG, and UFG specimens (deformed and undeformed) have also been corroborated by surface analyses after corrosion test. The SEM micrographs of the corroded surface are presented in Fig. 14 after a continuous 3-month immersion period in aerated saline solution. The corrosion resistance of stainless steel stems from its ability to readily passivate, forming protective films due to the presence of alloying elements (e.g. chromium and nickel)^36^. However, in chloride-enriched media, stainless-steel suffer corrosion due to unrestricted attack by aggressive chloride ions. Physical examination of the specimens’ surface after the 3-month exposure to saline solution revealed the occurrence of pitting corrosion since inherent defects in the passive layers allow for further dissolution of the material. The observed pits are localized at sites that are susceptible to chloride ion attack, especially if the anodic sites are widened. The observed extent of pitting corrosion varied, depending on the grain size of the steel as well as the rate of the prior-deformation (Fig. 14).

The results of the current study suggest that grain refinement has a significant influence on the mechanical strength and corrosion behavior of AISI 321 stainless steel (Figs 11 and 13). This is in agreement with the results obtained by other researchers^48–51^. The research findings in this study also align with those of another previous study on nanocrystalline structures^48^. Previous studies have also attributed the corrosion resistant of nanocrystalline stainless-steel structures to greater Cr diffusion/Cr-oxide layer^52–54^ and other factors associated with their enhanced mechanical properties^53–55^ rather than to the presence of coarse grains and alloying elements^36^. Factors associated with ultra-fine grain sizes and structural defects such as grain boundaries and triple points^56,57^, are considered in explaining the excellent corrosion resistance of nanocrystalline materials. The distribution of pits is relatively uneven and very localized while the passive films disallow further dissolution of the base metal. The morphology of pits varies depending on the grain size of the alloy. For the CG specimen that was deformed under quasi-static loading condition, pits are scattered in no definite pattern (Fig. 14c). The metastable pitting rate of the CG specimen must have significantly exceeded its repassivation ability, leading to the observed corrosion features. Surface pitting are deeper for the FG specimens (Fig. 14b,e,h) compared to its ultra-fine counterpart (Fig. 14a,d,g) especially along the TiN site. Also localized, the pit buildup is concentrated around the vicinity of TiN second-phase particles in the metal surface due to reduced chromium threshold concentration^58^. Across the fine matrix (also observed in UFG and CG structures), some of the TiN cubic particles are crushed, probably due to plastic deformation. It is also possible that the prolonged exposure of the specimens to the NaCl solution could be responsible for the crushed TiN cubic particles. Few pits observed along the δ-ferrite site (red arrows in Fig. 14). Fewer pitting sites and sizes are observed in the UFG specimens relative to the FG specimens. Corrosion is severe for the CG specimens compared to the FG specimens. According to Gupta and Birbilis^36^, increased metastable pitting rate could be attributed to elemental distribution (e.g. Mn and S) within the material and higher activity of nanocrystalline surface. The possibilities for metastable-stable pit’s transition in nanocrystalline stainless-steel materials is lower, hence, their rapid repassivation rate. The results obtained in this study reveal changes in electrochemical behaviour of metal due to grain refinement as a consequence of changing grain boundary densities^59^.

### Proposed pitting mechanisms for stainless-steel specimen in NaCl

**Figure 15:**
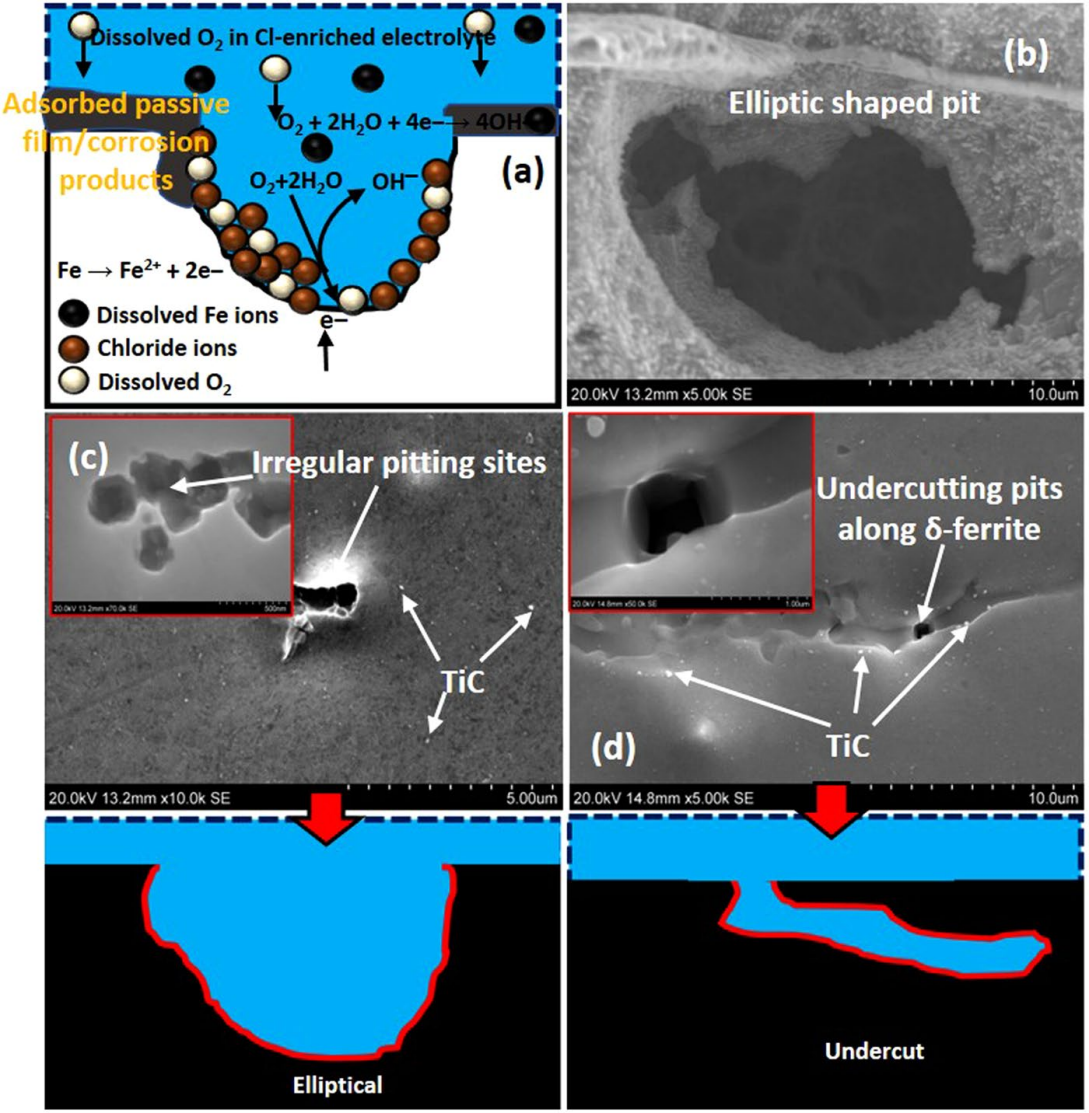
Pitting pattern at TiN sites of stainless-steel samples exposure to NaCl medium. This corrosion pattern is uniform for all samples studied in this work.

The pitting patterns in deformed and undeformed specimens are schematically presented in Fig. 15a. It is worthy of note that these metal samples exhibit varying corrosion behaviors, leading to rather complicated degradation mechanisms. Like most stainless-steel grades, the most prominent form of corrosion observed from SEM is pitting corrosion with clear evidence of corrosion surface pits. Combined actions of corrosive chloride ions and dissolved oxygen initiate these pits. Pitting persists when the rate of re-passivation is significantly slow, and the passivating films (cathodic) continuously leach out dissolved Fe ions from anodic metal surfaces (Fig. 15a). Subsurface pits are observed on undeformed UFG samples with shallow morphologies (Fig. 15b). Irregular elliptical-shaped pits are also observed on surfaces of UFG specimen that was previously subjected to quasi-static loading quasi-static condition (Fig. 15c), upon exposure to the corrosive medium. For UFG specimens, the influence of grain refinement on corrosion resistance could be linked to the metal’s ability to readily passivate, especially in environments to which passivity could be established^36,48^. The FG specimen that experienced dynamic loading condition before corrosion test exhibited undercutting pits that are observed at δ-ferrite site (Fig. 15d). The depassivation of these areas leads to intergranular corrosion due to insufficient chromium content. This work also confirms the non-detrimental effect of TiC particles to corrosion. SEM micrographs show that no pitting (or galvanic effect) occurred around TiC particles in the matrix and grain boundaries (Fig. 15c), and those forming necklace around the stringer ferrite (Fig. 15d).

**Figure 16:**
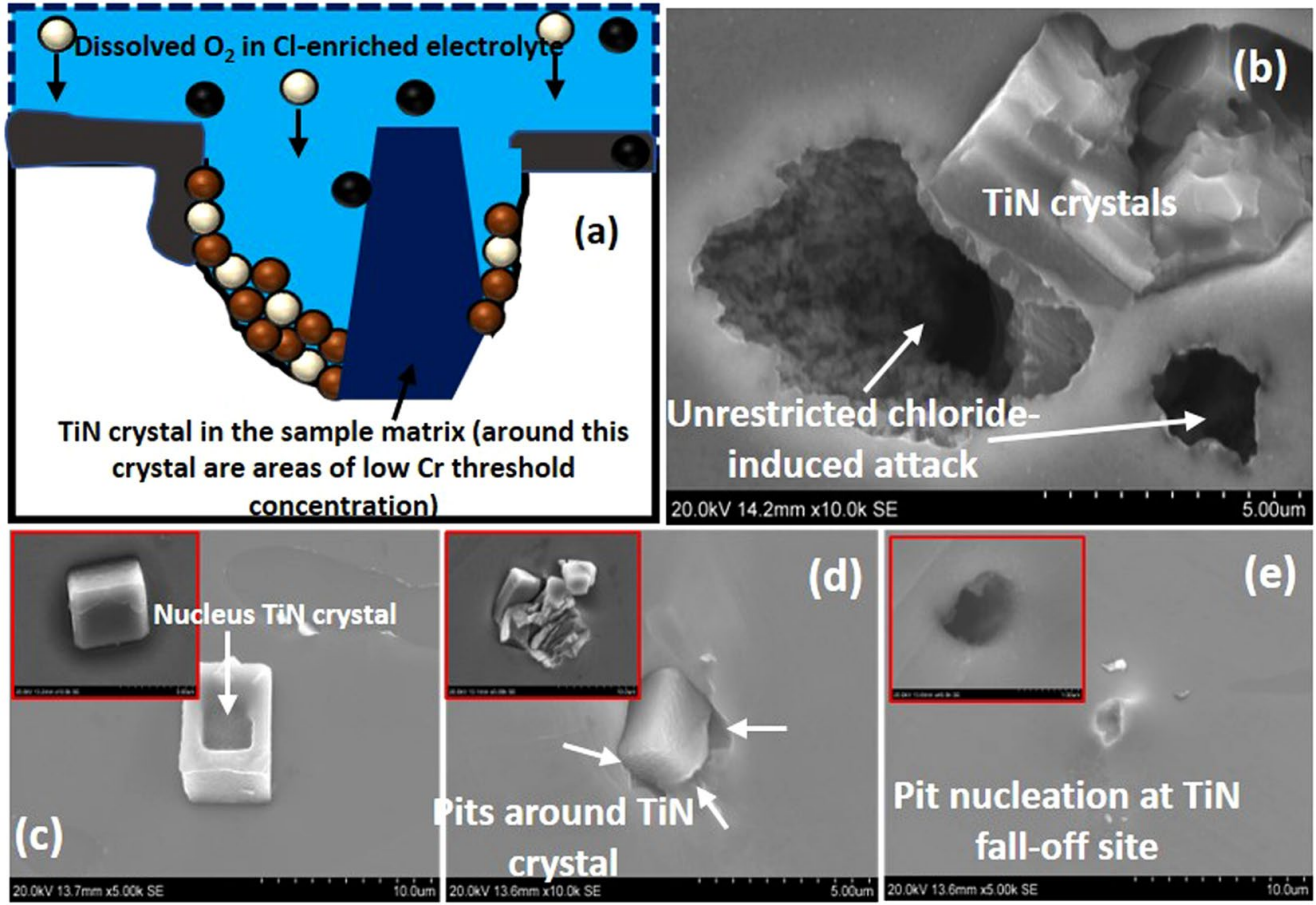
(**a**) Pitting pattern at TiN sites of stainless-steel samples exposure to NaCl medium, (**b**) Pit deepening around TiN sites due to reduced threshold chromium concentrations, (**c**) Smooth crystal showing some form of depression on its nucleus, (**d**) pit nucleation around TiN and (**e**) the early stages of pitting corrosion at the TiN fall-off site. This corrosion pattern is the same for all samples studied in this work; only the response of steel sample with coarse grains is presented. Inset: Micrographs of coarse grain specimens deformed under quasi-static condition.

Generally, austenitic stainless steels could be susceptible to thermal residual stress and sensitization. Sensitization defines the precipitation of chromium carbides around grain boundaries, leading to intergranular corrosion, and this is one of the foremost challenges encountered in welding of austenitic stainless steel apart from its low thermal conductivity. The introduction of carbide-formers (e.g. Ti) within AISI 321 creates a strong affinity for carbon (compared to Cr). This results in the formation of more stable solid TiC phases and leaves Cr in solution for corrosion protection^60^. The presence of secondary TiN phase is also recorded in the investigated AISI 321 stainless steel. Contrary to the type of pits presented in Fig. 15, the presence of TiN alters the corrosion dynamics. The morphologies of the TiN crystals embedded within the steel sample matrix could reasonably differ depending on the duration of the corrosion test as well as the type of pretreatment procedures utilized before imaging. Pit deepening are observed only around TiN sites for undeformed CG specimens compared to their ultra-fine counterpart (Fig. 16b). This could be attributed to the unrestricted chloride-induced corrosion attacks around these particles, especially where the threshold chromium concentrations are significantly low^58^. The dissolution of these TiN particles appears to be a gradual process. In Fig. 16, a previously smooth TiN particle within the bulk of the material now shows a pit around its nucleus (Fig. 16c). Another TiN site showing the onset of pitting is shown in Fig. 16d. Pit nucleation begins in a later formative stage and deepens to the subsurface. However, if the TiN particle falls off at the early stage of nucleation, perhaps, due to ultrasonic cleaning, the aggressive progression of pitting corrosion ceases as shown in Fig. 16e. This confirms that a galvanic coupling is usually set up between the TiN particle and its surrounding matrix^39^ and hence, resulting in the observed detrimental effect of TiN to pitting corrosion resistance, unlike the TiC particle. The corrosion resistance of AISI 321 stainless steel is therefore, not determined only by the ability to passivate, the presence of secondary stable phases can affect the rate of pitting corrosion.

## Conclusions

The effect of grain size, prior deformation and deformation rate on the corrosion behavior of AISI 321 austenitic stainless steel in 3.5 wt.% NaCl solution was determined. The as-received coarse-grained alloy (~37 µm grain size) was subjected to thermomechanical processing to develop fine (~3 µm grain size) and ultrafine (~0.24 µm grain size) grained structure. The coarse, fine and ultrafine grained specimens were deformed under dynamic (8.8 × 10^3^ s^−1^) and quasi-static (4.4 × 10^−3^ s^−1^) loading conditions to the same true strain of ~0.86 using a split Hopkinson pressure bar and Instron R5500 mechanical testing systems, respectively. Using XRD, SEM, TEM, EBSD, and EDS characterization techniques to investigate the steel specimens, the following conclusions are drawn;

- Corrosion resistance is highest in the UFG specimens, followed by FG and CG specimens, in that order. This is due to the formation of a more stable adsorbed passive film in UFG specimens.
- The corrosion resistance of AISI 321 austenitic stainless steel follows the order: quasi-static > dynamic > undeformed, for all grain sizes. This could be due to the evolution of close-packed crystallographic planes (CD||[110] for austenite and CD||[100] for martensite) that nullifies the adverse effect of DIM on the passivation and repassivation characteristics of AISI 321 steel compressed under quasi-static condition.
- The presence of TiC particles is not detrimental to the corrosion resistance of AISI 321 steel, whereas, galvanic coupling exist between TiN particles and their surrounding matrix leading to the pitting corrosion around TiN particles.
- Overall, the corrosion resistance of AISI 321 austenitic stainless steel is not determined only by the ability to passivate, the presence of secondary stable phases can affect the rate of pitting corrosion.

## Supplementary information

Supplementary info
