# Supplementary material for: Corrosion behavior of metastable AISI 321 austenitic stainless steel: Investigating the effect of grain size and prior plastic deformation on its degradation pattern in saline media
Source: Sci Rep. 2019 Aug 20;9:12116. doi: 10.1038/s41598-019-48594-3 (PMC6702197; doi:10.1038/s41598-019-48594-3)
Supplement: Supplementary file 1 — Supplementary info [file 41598_2019_48594_MOESM1_ESM.pdf]

Corrosion behavior of metastable AISI 321 austenitic stainless steel: Investigating the effect of grain size and prior plastic deformation on its degradation pattern in saline media

A.A. Tiamiyu*, Ubong Eduok**, J.A. Szpunar, A.G. Odeshi

Department of Mechanical Engineering, College of Engineering, University of Saskatchewan, 57 Campus Drive, Saskatoon, S7N 5A9, Saskatchewan, Canada

---

SUPPORTING INFORMATION

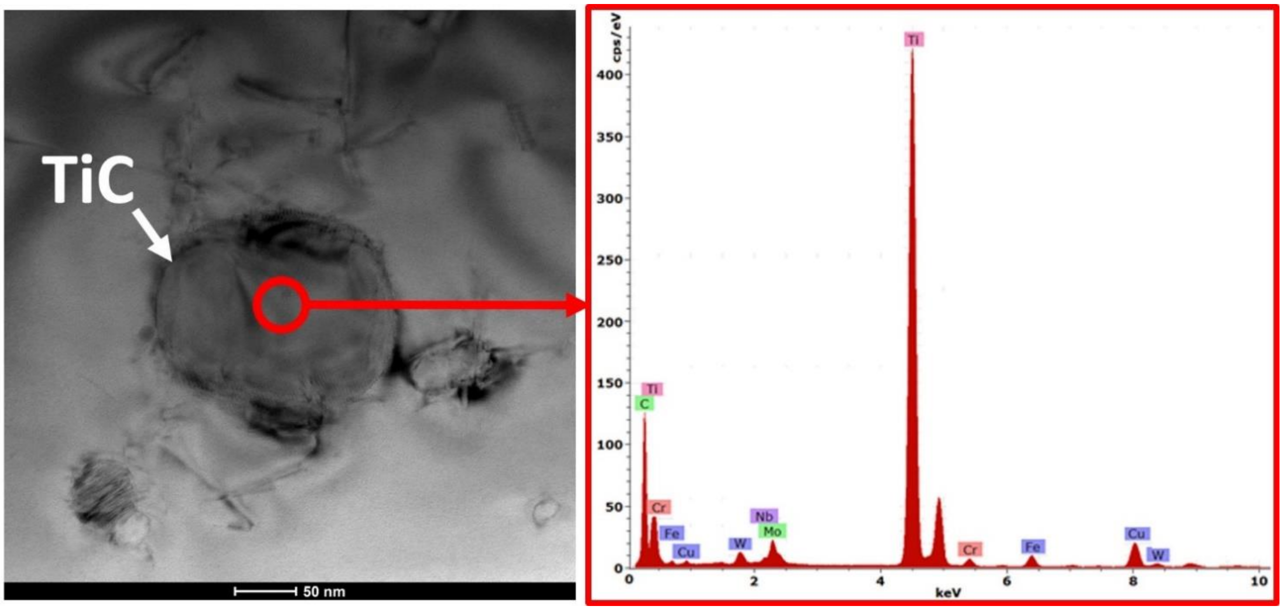

Fig. S1 TiC particle identified by STEM technique.

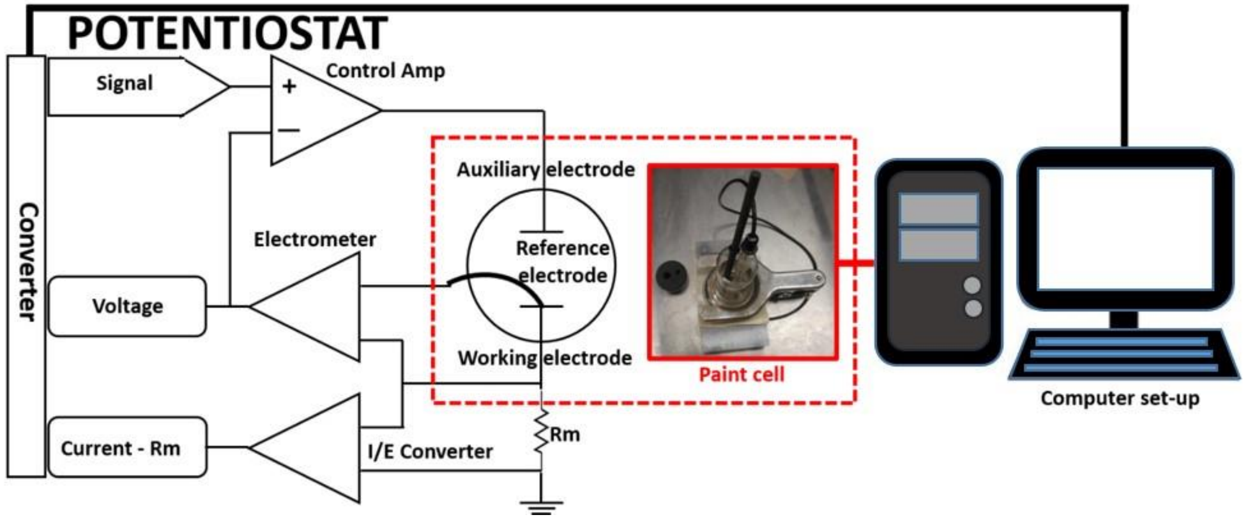

Fig. S2. Schematic representation of electrochemical set-up linked with a computer-controlled Gamry potentiostat system; the potentiostat schematic is adapted from Gamry webpage (https://www.gamry.com/application-notes/instrumentation/understanding-specs-of-potentiostat/)

Table S1. Electrochemical parameters for stainless-steel substrates with coarse, fine and ultra-fine grain sizes subjected to deformation at different strain rates as well their undeformed counterpart exposed to 3.5 wt.% NaCl solution at room temperature.

Study conditions (Grain size)	Substrates	Potentiodynamic polarization technique				Electrochemical impedance spectroscopy					
		β_a (mV/dec)	β_c (mV/dec)	j_{corr} (μA/cm^2)	$-E_{corr}$ (V vs SCE)	R_{soln} (Ω cm^2)	Q_{dl}, Y_o (μF cm^{-2} s$^{-(1-\alpha c)}$)	R_{ct} (Ω cm^2)	R_L (Ω cm^2)	L (H)	$\chi^2 \times 10^{-4}$
COARSE	Quasi-static	373.70	4.40	0.43	0.35	23.60	24.70	401.10	3642.0	152.50	745.500
	Dynamic	65.30	15.80	16.60	0.39	29.20	41.50	124.60	838.7	41.60	20.500
	Undeformed	73.90	706.80	142.90	0.60	23.50	68.10	83.10	0.015	35990.00	0.161
FINE	Quasi-static	461.80	298.90	0.39	0.35	24.70	24.20	609.20	646.1	12330.00	0.146
	Dynamic	284.10	244.20	2.95	0.53	21.10	26.50	822.40	2614	2195.00	0.411
	Undeformed	132.80	111.20	129.70	0.59	27.10	36.30	90.80	658	1331.20	0.359
ULTRA-FINE	Quasi-static	113.90	169.40	0.29	0.45	21.30	18.40	1509.00	5624	1.82	0.894
	Dynamic	28.80	249.50	0.59	0.36	25.40	23.70	1081.10	2896	24990.00	16.100
	Undeformed	40.30	55.50	16.30	0.29	27.30	48.40	175.00	856	465.80	0.251

Table S2. Electrochemical parameters for both undeformed and deformed stainless-steel substrates with varying grain sizes exposed to 3.5 wt.% NaCl solution at room temperature.

Study conditions (Deformation)	Substrates	Potentiodynamic polarization technique				Electrochemical impedance spectroscopy					
		β_a (mV/dec)	β_c (mV/dec)	j_{corr} (μA/cm^2)	$-E_{corr}$ (V vs SCE)	R_{soln} (Ω cm^2)	Q_{dl}, Y_o (μF cm^{-2} s$^{-(1-\alpha c)}$)	R_{ct} (Ω cm^2)	R_L (Ω cm^2)	L (H)	$\chi^2 \times 10^{-4}$
UNDEFORMED	Ultra-fine	40.30	55.50	16.30	0.29	27.30	48.40	175.00	856	465.80	0.251
	Fine	132.80	111.20	129.70	0.59	27.10	36.30	90.80	658	1331.20	0.359
	Coarse	73.90	706.80	142.90	0.60	23.50	68.10	83.10	0.015	35990.00	0.161
DYNAMIC	Ultra-fine	28.80	249.50	0.59	0.36	25.40	23.70	1081.10	2896	24990.00	16.100
	Fine	284.10	244.20	2.95	0.53	21.10	26.50	822.40	2614	2195.00	0.411
	Coarse	65.30	15.80	16.60	0.39	29.20	41.50	124.60	838.7	41.60	20.500
QUASI-STATIC	Ultra-fine	113.90	169.40	0.29	0.45	21.30	18.40	1509.00	5624	1.82	0.894
	Fine	461.80	298.90	0.39	0.35	24.70	24.20	609.20	646.1	12330.00	0.146
	Coarse	373.70	4.40	0.43	0.35	23.60	24.70	401.10	3642.0	152.50	745.500
